# Regeneration of toxigenic *Pasteurella multocida* induced severe turbinate atrophy in pigs detected by computed tomography

**DOI:** 10.1186/1746-6148-9-222

**Published:** 2013-10-30

**Authors:** Tibor Magyar, Tamás Donkó, Imre Repa, Melinda Kovács

**Affiliations:** 1 Institute for Veterinary Medical Research, Centre for Agricultural Research, Hungarian Academy of Sciences, Budapest, Hungary; 2 Institute of Diagnostic Imaging and Radiation Oncology, Kaposvár University, Kaposvár, Hungary; 3 Department of Physiology and Animal Hygiene, Faculty of Animal Science, Kaposvár University, Kaposvár, Hungary

**Keywords:** Atrophic rhinitis, *Bordetella bronchiseptica*, *Pasteurella multocida*, Computed tomography

## Abstract

**Background:**

Atrophic rhinitis is a widely prevalent infectious disease of swine caused by *Bordetella bronchiseptica* and *Pasteurella multocida*. The course of the disease is considered to be different depending on the principal aetiological agents distinguishing *B*. *bronchiseptica* induced non-progressive and toxigenic *P*. *multocida* produced progressive forms. In order to compare the pathological events of the two forms of the disease, the development of nasal lesions has longitudinally been studied in pigs infected by either *B*. *bronchiseptica* alone or *B*. *bronchiseptica* and toxigenic *P*. *multocida* together using computed tomography to visualise the nasal structures.

**Results:**

*B*. *bronchiseptica* infection alone caused moderately severe nasal turbinate atrophy and these lesions completely regenerated by the time of slaughter. Unexpectedly, complete regeneration of the bony structures of the nasal cavity was also observed in pigs infected by *B*. *bronchiseptica* and toxigenic *P*. *multocida* together in spite of seeing severe turbinate atrophy in most of the infected animals around the age of six weeks.

**Conclusions:**

*B*. *bronchiseptica* mono-infection has been confirmed to cause only mild to moderate and transient lesions, at least in high health status pigs. Even severe turbinate atrophy induced by *B*. *bronchiseptica* and toxigenic *P*. *multocida* combined infection is able to be reorganised to their normal anatomical structure. Computed tomography has further been verified to be a useful tool to examine the pathological events of atrophic rhinitis in a longitudinal manner.

## Background

Atrophic rhinitis (AR) is a widespread infectious disease of swine characterised by twisting or shortening of the snout [1]. This facial deformity results from an underlying atrophy of the nasal turbinate bones, which is the principal pathological lesion of AR. Two infectious agents are associated with the aetiology of AR: toxigenic strains of *Bordetella bronchiseptica* and *Pasteurella multocida*. A heat-labile toxin of *P*. *multocida* (PMT) plays a dominant role in developing the characteristic lesions of AR, and so toxigenic *P*. *multocida* is considered to be the primary aetiological agent of the disease. On the other hand, a substantial body of evidence shows that *P*. *multocida* is unable to fulfil its role without predisposing circumstances of which *B*. *bronchiseptica* pre-infection is the most commonly recognised [2,3]. Because of this specific synergistic interaction between *B*. *bronchiseptica* and *P*. *multocida* AR is classified as a genuine polymicrobial disease [4].

Although both *B*. *bronchiseptica* and *P*. *multocida* have the ability to cause turbinate atrophy in pigs, a number of research findings show that the severity and persistence of the pathological changes generated by these two bacteria are different. *B*. *bronchiseptica* infection has been reported to result in mild-to-moderate lesions that could regenerate by the time pigs reach slaughter weight [5,6], while toxigenic strains of *P*. *multocida* proved to be able to produce severe and irreversible turbinate atrophy [2,7]. These observations led to the proposition to distinguish non-progressive and progressive forms of AR according to the principal aetiological agents that can be found in the herd in question [8]. Nevertheless, no exact description of the course of the disease from infection in a young age until reaching slaughter weight is available in the literature.

In early longitudinal studies of AR, large numbers of pigs were used because several animals had to be euthanized at selected time-points in order to obtain a satisfactory number of cross-sectional snout slices to perform the evaluations. Moreover, this approach provided no data about the changes in individual animals over time. These problems were overtaken by the introduction of computed tomographic imaging that allowed non-invasive tracking of the pathogenesis of AR within single pigs examining them at several time-points without the need to sacrifice their life [9-11].

The purpose of this study was to follow the morphological changes of nasal structures in pigs infected by either *B*. *bronchiseptica* alone or *B*. *bronchiseptica* and toxigenic *P*. *multocida* together using computed tomography (CT) for visualising the nasal cavities of the experimental animals.

## Methods

### Experimental animals

The experiments complied with current Hungarian legislation and were authorised by the Food Chain Safety and Animal Health Directorate of the Somogy County Agricultural Office (Ref: 406-6/2002) based on the approval of the Ethics Committee of the Ministry of Agriculture.

Twenty-eight new-born piglets from a single closed herd were selected for each experiment. Clinical signs of AR had never been observed in the herd and toxigenic *P*. *multocida* had never been identified. No vaccination against AR had ever been undertaken on the farm. The *P*. *multocida*-free status of the selected animals was confirmed prior to initiating the study. Medicated early weaning was applied to obtain *B*. *bronchiseptica*-free piglets. The sows delivering the piglets had been treated with tulathromycin (Draxxin inj., Pfizer, New York, USA) administered in a dose of 1 ml/40 kg one week before farrowing. The piglets were moved to isolated units at 1–2 days of age where they were kept in heated compartments. Pigs were fed with Sprayfo milk replacer (Sloten, Deventer, Netherlands) until the age of 28 days using Mambo milk applicator device (Sloten, Deventer, Netherlands) according to the manufacturer’s recommendations. At the age of 28 days the piglets were weaned from milk replacer and then consumed only prestarter feed. From 6 to 10 weeks of age they were fed with starter diet, and then fattening diet until the termination of the study. Animals were fed ad libitum from self-feeders and had free access to drinking water. No treatments were permitted that could have interfered with the outcome of the trial, such as the use of immunosuppressive drugs or antibiotics affecting the respiratory tract.

### Bacterial strains

Toxigenic strain KM22 of *B*. *bronchiseptica* was isolated from a herd with clinical AR. LFB3, a toxigenic isolate of *P*. *multocida*, was kindly supplied by Dr JM Rutter (Institute for Animal Health, Compton, UK). Both strains are known to produce the disease [12]. *B*. *bronchiseptica* strain KM22, cultured on Bordet–Gengou (BG) agar (Oxoid, Basingstoke, UK) at 37C for 24 hours, was diluted in phosphate-buffered saline (PBS), pH 7.2, to give a suspension of about 10^6^ Colony Forming Units/ml (CFU/ml). *P*. *multocida* strain LFB3, cultured on 5% sheep blood agar (BA) at 37C for 24 hours, was diluted in Brain Heart Infusion (BHI) broth (Difco, Detroit, USA) to give a suspension of about 10^8^ CFU/ml. The toxin-producing ability of *P*. *multocida* strain LFB3 was confirmed by a membrane assay [13].

### Experimental design

Two experiments were performed. Each experiment consisted of two groups of 14 randomly selected piglets. Group 1 piglets were infected with either *B*. *bronchiseptica* alone or *B*. *bronchiseptica* and *P*. *multocida* together. Group 2 piglets remained uninfected (negative) controls. The day of *B*. *bronchiseptica* infection was study day 0 (D0) of the study.

Piglets in Group 1 in Experiment 1 were infected with *B*. *bronchiseptica* at 4 days of age by instilling 0.5 ml of bacterial suspension into each nostril. Piglets in Group 1 in Experiment 2 were infected with *B*. *bronchiseptica* at 4 days of age by instilling 0.5 ml of bacterial suspension into each nostril, and with *P*. *multocida* at 8 days of age by the same method.

Experiment 1 was terminated at 132 days of age and Experiment 2 at 128 days of age. At termination, the pigs were slaughtered and examined in a blind manner for turbinate bone atrophy (TA), nasal septum deviation (NSD) and lung lesions.

### Collection and bacteriologic examination of nasal secretions

Nasal swabs were taken from piglets at each CT examination. The samples were tested for the presence of *B*. *bronchiseptica* on MacConkey agar (BBL, Sparks, USA) as described elsewhere [14]. The presence of *P*. *multocida* toxin in the primary cultures of nasal swabs was determined using a PMT ELISA (Oxoid, Hampshire, UK) according to the manufacturer’s recommendation.

### Computer tomography

To immobilise the pigs for the scanning procedure, 4 mg/kg azaperone (Stresnil, Janssen Pharmaceutica) was administered intramuscularly for sedation and 9 mg/kg ketamine hydrochloride (SBH-Ketamin inj., SelBruHa) was administered intravenously to anaesthetise the pigs.

The anaesthetised pigs were fixed in stretched position, lying flat in a purpose-designed container during the CT examination. CT images from each pig were acquired using a Somatom Plus 40 (Siemens, Erlangen, Germany) third generation scanner. The starting position of the scanning was set approximately 10 mm anterior from the level of the first upper premolar teeth, and then consecutive three mm scans were taken with a table feed of five mm. The imaging protocol was a so-called “high” algorithm, which is extremely sensitive in visualising tissues of high-density differences. Zoom factor was set to 3.5.

In Experiment 1, CT images of all pigs were acquired at study days 0, 9, 41, 70, 101, and 132 while in Experiment 2 they were obtained at study days 0, 4, 18, 25, 32, 60, 88, and 128. Non-infected pigs were always imaged prior to the infected pigs to avoid cross-contamination.

### Interpretation of CT images

Visual scoring of the nasal turbinate bones was done on CT scans at the level of the first premolar teeth. Each of the four scrolls of the ventral turbinate bones was scored according to the following criteria (TA score): 0, no lesion; 1, a small part of the turbinate bone (nearly half a scroll) is absent; 2, slight atrophy – more than half a scroll is absent; 3, moderate atrophy – the turbinate bone is straightened; 4, severe atrophy – total disappearance of the turbinate bone. NSD was scored on a scale of 0–2: 0, normal; 1, slight deviation; 2, severe deviation. TA and NSD scores were summed for each individual to a maximum value of 18 (nasal lesion score, NLS).

## Results

### Animal health and bacteriology

*B*. *bronchiseptica* infected piglets did not show noteworthy clinical signs: only sporadic sneezing, mild nasal discharge and lacrimation were observed in some of the animals. In Experiment 2, *B*. *bronchiseptica* infection produced the same results while pronounced sneezing was noticed in all infected piglets from 2 days after the *P*. *multocida* infection. It lasted approximately one week then gradually disappeared.

Three pigs were euthanized for welfare reasons because of emaciation and weakness during their first weeks of life. No connection was established between these signs and the experimental infection.

*B*. *bronchiseptica* infected animals (Experiment 1) produced *B*. *bronchiseptica* culture positive samples up to D41 post infection. Most of the *B*. *bronchiseptica* - *P*. *multocida* infected animals (Experiment 2) yielded *B*. *bronchiseptica* positive samples until D60. In the control pigs neither culture positive *B*. *bronchiseptica* isolation nor PMT identification was produced from their samples. The *B*. *bronchiseptica* infected pigs (Experiment 1) were also PMT negative throughout the whole observation period. PMT monitoring of the *P*. *multocida* infected pigs during each imaging did not result in consistent detection of PMT (Table 1).

**Table 1:** **Detection of P**. **multocida toxin** (**PMT**) **in piglets in group 1 in experiment 2**

**Pig ID**	**Post infection day**							
	**0**	**4**	**18**	**25**	**32**	**60**	**88**	**128**
4	-	-	+	-	+	-	-	-
5	-	-	+	+	-	-	-	-
9	-	-	+	+	-	-	-	-
12	-	-	-	+	+	-	-	-
13	-	-	-	+	+	-	-	-
16	-	-	-	+	-	+	-	-
19	-	-	+	-	+	-	-	-
21	-	-	+	-	+	+	-	-
22	-	-	+	+	+	-	-	-
25	-	-	-	+	-	+	-	-
27	-	-	-	+	+	-	-	-
29	-	-	+	-	+	-	-	-
30	-	-	-	+	+	-	-	-
32	-	-	+	-	-	-	-	-

The piglets were infected intranasally with *B*. *bronchiseptica* at the age of 4 days (D0) and *P*. *multocida* at the age of 8 days (D4). PMT was shown out with a PMT ELISA (Oxoid, Hampshire, UK).

### Assessment of CT images

No pathological alterations of the nasal structures were noticed in the uninfected control pigs.

**Figure 1:**
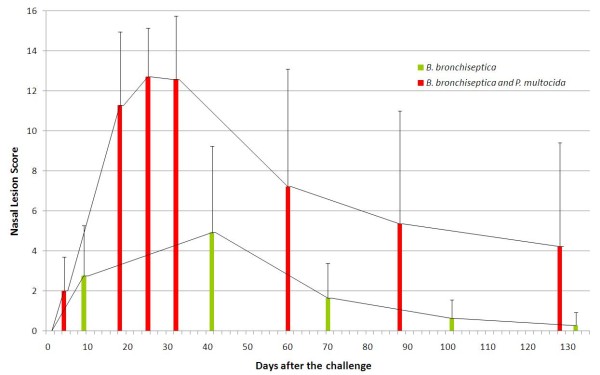
**Development of nasal lesion scores in the infected piglets.** The piglets were infected with either *B*. *bronchiseptica* alone (Group 1, Experiment 1) or *B*. *bronchiseptica* and *P*. *multocida* together (Group 1, Experiment 2). The diagram shows the mean nasal lesions scores calculated from the individual scores of the computed tomography scans gained throughout the observation period.

In both infected groups (Experiment 1 and 2), regressive changes were observed in the nasal architecture (Tables 2 and 3). However, the progression of these changes showed great individual variability. *B*. *bronchiseptica* infected pigs produced lower scores and greater diversity of the lesions than the pigs in the *B*. *bronchiseptica* - *P*. *multocida* infected group in which more even lesions and higher scores were recorded (Figure 1).

**Table 2:** Nasal lesion scores of piglets in group 1 in experiment 1

**Pig ID**	**Post infection day**					
	**0**	**9**	**41**	**70**	**101**	**132**
1	0	8	Died			
2	0	Died				
3	0	Died				
4	0	3	3	2	1	0
5	0	3	3	0	0	0
6	0	1	1	0	0	0
7	0	0	12	5	1	0
9	0	4	11	3	1	0
10	0	3	8	4	3	2
13	0	0	2	1	0	0
16	0	0	0	1	0	0
21	0	6	8	2	1	1
22	0	1	0	0	0	0
24	0	4	6	0	0	0
Means	0	2,8	4,9	1,6	0,6	0,3

The piglets were infected intranasally with *B*. *bronchiseptica* at the age of 4 days (D0).

**Table 3:** Nasal lesion scores of piglets in group 1 in experiment 2

**Pig ID**	**Post infection day**							
	**0**	**4**	**18**	**25**	**32**	**60**	**88**	**128**
4	0	2	12	15	15	3	2	0
5	0	3	12	12	10	0	0	1
9	0	0	2	6	6	1	1	0
12	0	0	11	14	13	12	12	7
13	0	0	6	8	7	1	0	0
16	0	1	13	13	14	11	8	9
19	0	1	15	14	14	9	3	3
21	0	2	13	15	15	15	11	11
22	0	3	14	14	16	17	18	16
25	0	3	14	14	16	8	5	9
27	0	4	14	14	14	5	3	4
29	0	5	9	12	12	4	5	3
30	0	4	14	14	14	15	11	6
32	0	0	9	12	10	1	0	0
Means	0,0	2,0	11,3	12,6	12,6	7,3	5,6	4,9

The piglets were infected intranasally with *B*. *bronchiseptica* at the age of 4 days (D0) and *P*. *multocida* at the age of 8 days (D4).

**Figure 2:**
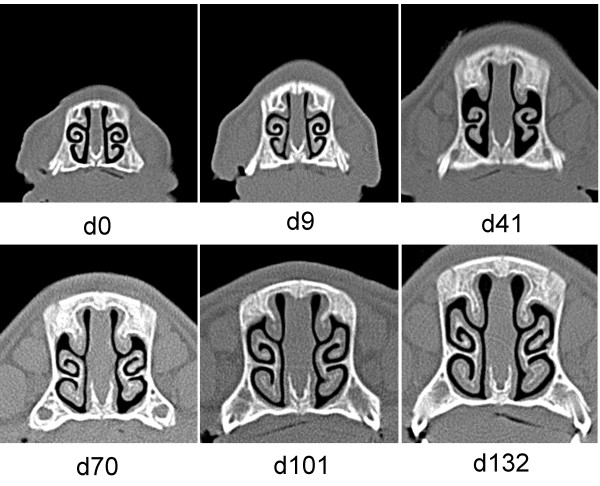
**Sequential computed tomography scans of pig No. 7 in Experiment 1.** Pig No. 7 was infected with *B*. *bronchiseptica* at the age of 4 days (D0). This pig showed the most severe nasal turbinate atrophy (nasal lesion score 12) in this group that completely regenerated until Day 101 of the study.

Only 4 out of 11 live piglets had 8 or higher NLS in the *B*. *bronchiseptica* infected group on D41 of the study. The lesions started to quickly regenerate after D41 and all animals became practically free of lesions until D101, i.e. the end, of the study. Figure 2 shows a pig having the highest NLS in this group at D41 and producing complete regeneration of the lesion by the time of termination.

**Figure 3:**
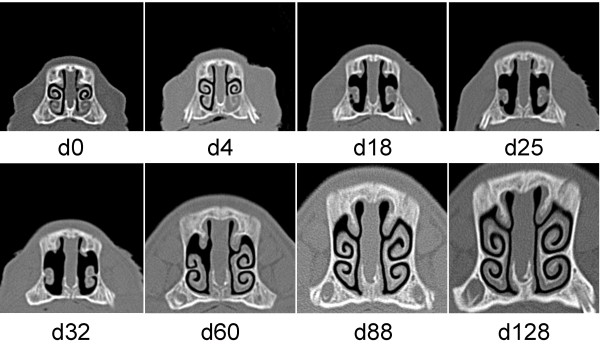
**Sequential computed tomography scans of pig No. 4 in Experiment 2.** Pig No. 4 was infected with *B*. *bronchiseptica* at the age of 4 days (D0) and *P*. *multocida* at the age of 8 days (D4). Complete regeneration of the nasal turbinates was observed although severe turbinate atrophy (nasal lesion score 15) was seen from Day 18 to Day 32.

**Figure 4:**
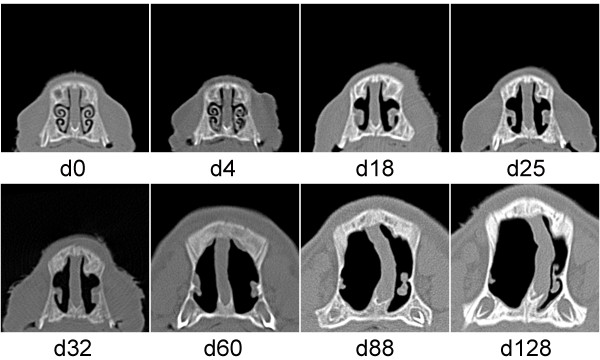
**Sequential computed tomography scans of pig No. 22 in Experiment 2.** Pig No. 22 was infected with *B*. *bronchiseptica* at the age of 4 days (D0) and *P*. *multocida* at the age of 8 days (D4). Severe turbinate atrophy and nasal septum deviation (nasal lesion score 16–18) developed that progressed throughout the whole observation period.

In the *B*. *bronchiseptica* - *P*. *multocida* infected group, 12 out of the 14 piglets had 12 or higher NLS by the time of D25 indicating rapidly developing and severe turbinate atrophy in the whole group. Interestingly, most of the pigs showed unexpected signs of reparation noticed from D60 and at the time of the termination of the study (D128) 7 out of the 14 pigs had a score of ≤3. Figure 3 demonstrates an example of such a complete regeneration of turbinate atrophy with a score of 15 on D25 and 32. Figure 4 illustrates the only case when the severe TA and NSD lasted until the termination of the study.

## Discussion

Our results confirmed the suggestion by a number of authors [2,5,6,15] that *B*. *bronchiseptica* infection alone causes only moderately severe atrophy of the nasal turbinate bones and these lesions can completely regenerate over time. The pathological changes produced in the present study progressed until about 6 weeks of age, and then remarkable recovery was seen 30 days later followed by complete healing by the time of slaughter.

Former *B*. *bronchiseptica* challenge experiments showed conflicting results as to the degree of turbinate atrophy found in the infected pigs. One possible explanation could have been that variations in virulence of the various *B*. *bronchiseptica* strains may influence the outcome of the infection. However, Rutter et al. [16] could not demonstrate such differences among *B*. *bronchiseptica* strains independently whether they had been isolated from herds with or without AR. It seems more likely that the severity of the lesions is influenced by the health status of the piglets used in the study. In caesarean-derived, colostrum-deprived pigs [17] or gnotobiotic piglets [14], *B*. *bronchiseptica* infection resulted in moderate turbinate atrophy while severe lesions were developed in AR free but otherwise conventional piglets using the same *B*. *bronchiseptica* strain (B58) [18]. Conventional piglets might be more susceptible to *B*. *bronchiseptica* or other factors of the porcine respiratory disease complex may be involved in the disease process and promote the pathological effect exerted by *B*. *bronchiseptica* infection. We also used high health status animals in this study obtained by a method that followed the principles described by Alexander et al. [19] that may explain the low nasal lesion scores detected in the infected piglets.

It is generally accepted that toxigenic *P*. *multocida* causes irreversible nasal choncal hypoplasia. Therefore, it was a rather unexpected finding that even complete regeneration of the bony structures of the nasal cavity could be observed in pigs which had received a *B*. *bronchiseptica* and toxigenic *P*. *multocida* combined challenge in spite of the fact that severe turbinate atrophy was seen in most of the infected animals at 25 and 32 days after the first infection. Although bacteriological examination has not clearly verified the persistent colonisation of the nasal mucosa by toxigenic *P*. *multocida*, the success of the combined infection has been indicated by the development of these characteristic lesions. Thirty days later, however, significant reparation of the lesions was seen in 9 out of the 14 infected pigs. At the same time, in one pig the characteristic degree of progressive turbinate atrophy and severe nasal septum deviation persisted until termination of the observation period. No differences in any examined parameter, including bacteriological findings, could be found in this pig to explain why the lesions progressed until the end of the experiment.

It is also remarkable that, on average, the highest degree of turbinate atrophy has been detected around the age of six weeks in both infected groups which is the conventional time for nasal lesion scoring in vaccine efficacy studies performed according to the requirements of the European Pharmacopoeia. It means that the timing of the evaluation is the most probable stage at which the lesions reach their maximum degree, and thus it appears to be optimal for measuring the effectiveness of AR vaccines. On the other hand, it seems questionable how the disease would proceed after this age that may be worth further studies to clarify its impact on the assessment of the protective value of the vaccines.

Previously, Jolie et al. [9] who first applied CT for the diagnosis of porcine AR reported a gradual decline in nasal lesion score occurred in one pig selected from a commercial herd with clinically apparent atrophic rhinitis examined from three weeks of age until slaughter. However, neither *P*. *multocida* nor *B*. *bronchiseptica* was isolated from nasal swab samples and thus the exact aetiology and the form of AR represented by this case could not be established. Therefore, this is the first adequately supported demonstration that nasal turbinate bones may regenerate even from a stage of full score atrophy characteristic for the progressive form of AR. It also means that the definition of progressive AR may need some reconsideration. Our results strongly suggest that a heavy infection with PMT producing *P*. *multocida* assisted by previous *B*. *bronchiseptica* infection is not necessarily sufficient for the induction of severe and irreversible turbinate atrophy seen in conventional pigs examined at slaughter from AR positive herds. AR as a part of the porcine respiratory disease complex has been categorised as a multifactorial disease in which the two well-identified pathogens (*B*. *bronchiseptica* and toxigenic *P*. *multocida*) are working together with a number of predisposing factors like additional infectious agents, housing problems or poor management. Further studies are needed to recognise other factor or factors of possible importance and to understand their role in the aetiology and pathogenesis of AR.

CT has proved to be a feasible tool for such investigations. The unceasing development of CT equipments have resulted in continuously improving image resolution that, together with increasing scanning speed, makes possible the examination of smaller and smaller details. Its ability to follow the course of the disease in the same animal without the need to sacrifice is of great importance as well as its potential application for studying other diseases in a longitudinal manner just as proved recently by early detection of pneumonia and monitoring the pathological events during its progression [20,21].

## Conclusions

The transient nature of *B*. *bronchiseptica* induced nasal turbinate lesions was confirmed in a longitudinal manner. *B*. *bronchiseptica* infection alone caused only mild to moderate lesions that completely regenerated over time, at least in the high health status pigs used in the study. Although *B*. *bronchiseptica* and toxigenic *P*. *multocida* combined infection induced severe turbinate atrophy, even these lesions were able to be reorganised to their normal anatomical structure suggesting that the pathogenesis of the progressive irreversible form of AR maybe more complicated than it has been assumed so far. CT is a very valuable device for a non-invasive tracking of the pathological events of AR examining the same animal at several occasions.

## Competing interests

The authors declare that they have no competing interests.

## Authors’ contributions

TM conceived the study design, performed bacteriological procedures, participated in experimental work, data analysis and drafted the manuscript. TD had important input into experimental work, data collection and data analysis. IR coordinated the computed tomography examinations, contributed to data collection and data analysis. MK participated in working out the study design, coordinated the work and commented on the manuscript. All authors read and approved the final version of the manuscript.
